# Diagnostic accuracy of whole heart coronary magnetic resonance angiography: a systematic review and meta-analysis

**DOI:** 10.1186/s12968-023-00949-6

**Published:** 2023-06-26

**Authors:** Shingo Kato, Mai Azuma, Naoki Nakayama, Kazuki Fukui, Masanori Ito, Naka Saito, Nobuyuki Horita, Daisuke Utsunomiya

**Affiliations:** 1 Department of Diagnostic Radiology, Yokohama City University Graduate School of Medicine, Yokohama, Japan; 2 Department of Cardiology, Kanagawa Cardiovascular and Respiratory Center, Yokohama, Japan; 3 Department of Clinical Laboratory, Kanagawa Children’s Medical Center, Yokohama, Japan; 4 Chemotherapy Center, Yokohama City University Graduate School of Medicine, Yokohama, Japan

**Keywords:** Magnetic resonance angiography, Coronary artery disease, Diagnostic accuracy, Meta-analysis

## Abstract

**Background:**

The purpose of this meta-analysis was to comprehensively investigate the diagnostic ability of 1.5 T and 3.0 T whole heart coronary angiography (WHCA) to detect significant coronary artery disease (CAD) on X-ray coronary angiography.

**Methods:**

A literature search of electronic databases, including PubMed, Web of Science Core Collection, Cochrane advanced search, and EMBASE, was performed to retrieve and integrate articles showing significant CAD detectability of 1.5 and 3.0 T WHCA.

**Results:**

Data from 1899 patients from 34 studies were included in the meta-analysis. 1.5 T WHCA had a summary area under ROC of 0.88 in the patient-based analysis, 0.90 in the vessel-based analysis, and 0.92 in the segment-based analysis. These values for 3.0 T WHCA were 0.94, 0.95, 0.96, respectively. Contrast-enhanced 3.0 T WHCA had significantly higher specificity than non-contrast-enhanced 1.5 T WHCA on a patient-based analysis (0.87, 95% CI 0.80–0.92 vs. 0.74, 95% CI 0.64–0.82, P = 0.02). There were no differences in diagnostic performance on a patient-based analysis by use of vasodilators, beta-blockers or between Asian and Western countries.

**Conclusions:**

The diagnostic performance of WHCA was deemed satisfactory, with contrast-enhanced 3.0 T WHCA exhibiting higher specificity compared to non-contrast-enhanced 1.5 T WHCA in a patient-based analysis. There were no significant differences in diagnostic performance on a patient-based analysis in terms of vasodilator or beta-blocker use, nor between Asian and Western countries. However, further large-scale multicentre studies are crucial for the widespread global adoption of WHCA.

**Supplementary Information:**

The online version contains supplementary material available at 10.1186/s12968-023-00949-6.

## Background

Coronary artery disease (CAD) is a primary cause of mortality in the United States, and ranks as the third most common cause of death globally, responsible for 17.8 million deaths annually [1]. X-ray coronary angiography is utilized to diagnose CAD; however, it is an invasive procedure, and its complications cannot be overlooked. Presently, coronary computed tomography (CT) is widely employed in clinical practice as a non-invasive examination method. Coronary artery CT boasts a high negative predictive value and is efficacious in ruling out CAD [2]. Furthermore, there is evidence that evaluating coronary plaque [3] and implementing CT-based strategies can enhance prognosis [4]. Additionally, cost-effectiveness is also favorable in low to moderate prevalence rates [5]. Despite the utility of coronary CT being extremely high, it does have several drawbacks including radiation exposure, the administration of contrast agent, and difficulties in utilizing the method for highly calcified coronary arteries.

Whole heart coronary magnetic resonance angiography (WHCA) is considered as an alternative to coronary CT, possessing advantages over CT such as no radiation exposure, less susceptibility to coronary calcification [6]. Prior meta-analyses have been conducted on the diagnostic capabilities of WHCA [7, 8]. However, it should be noted that non-contrast imaging is recommended for 1.5 T WHCA, whereas contrast imaging is recommended for 3.0 T WHCA. The rationale behind this recommendation is that in 1.5 T WHCA, it is difficult to achieve increased arterial contrast with the administration of contrast agents [9]. In contrast, in 3.0 T WHCA, the use of steady state free precession (SSFP) is challenging due to banding artifacts caused by specific absorption ratio (SAR) limitations and B1 inhomogeneity. Therefore, it is generally advised to use gadolinium based contrast agents in gradient echo (GRE) sequence for 3.0 T WHCA [10]. To date, no meta-analysis has compared the diagnostic accuracy of non-contrast 1.5 T WHCA and contrast-enhanced 3.0 T WHCA. There is also debate about the need for premedication (vasodilators and beta-blockers) prior to imaging and differences in utilization by region (Western vs. Asian countries). These issues have not been evaluated in prior meta-analyses. Therefore, the purpose of this study was to perform a comprehensive meta-analysis on the diagnostic accuracy of WHCA for detecting significant CAD on X-ray coronary angiography and to evaluate the differences in magnetic field strength and use of contrast agent, with and without premedication, and including differences in diagnostic accuracy by region.

## Methods

A systematic literature search was conducted in accordance with the guidelines established by the Cochrane Collaboration and the Preferred Reporting Items for Systematic Review and Meta-analysis (PRISMA) on November 10, 2022, utilizing databases such as PubMed, Web of Science Core Collection, Cochrane advanced search, and EMBASE. Search terms utilized included “whole heart coronary magnetic resonance angiography”, “WHCA”, “MRI”, “coronary artery disease”, “diagnostic accuracy” (as outlined in Additional file 1: Material S1). Two evaluators (SK and MA) independently assessed the validity of all titles and abstracts, followed by a review of the relevant complete peer-reviewed studies; any discrepancies were resolved by a third reviewer. The protocol for this study was registered with the University Medical Information Network (registration number: UMIN000050172) and did not require institutional review board approval as it was a meta-analysis and did not involve clinical patient information. Both prospective and retrospective studies that included diagnostic performance of coronary WHCA at 1.5 T and 3.0 T for detecting significant CAD on X-ray coronary angiography were included for data extraction, while literature such as case reports, animal studies, and non-English language articles were excluded.

### Outcome measures

The primary objective of this meta-analysis was to estimate the diagnostic performance of coronary WHCA for significant coronary artery stenosis in known or suspected CAD using X-ray coronary angiography as the gold standard and to compare its value at 1.5 T and 3.0 T. Two reviewers (SK and MA) were invited to review the results of the studies, extracting the following study characteristics: author name, year of publication, country, patient disease, age, gender, magnetic resonance imaging (MRI) parameters such as magnetic field strength, sequence used, producer of MRI equipment, MRI coil information, use of gadolinium contrast, and examination time. Definition of significant CAD on X-ray coronary angiogram was also investigated. A meta-analysis of the diagnostic accuracy of coronary WHCA for significant coronary artery stenosis was performed using summary receiver operating characteristics (ROC) analysis. The analysis included the following. (1) All studies including the diagnostic performance of 1.5 T and 3.0 T were used to compare their diagnostic performance. The following sub-analyses were performed: 1. non-contrast enhanced 1.5 T WHCA vs. contrast enhanced 3.0 T WHCA, 2. drug administration (vasodilators and beta-blockers), 3. Comparison between Asian and Western countries. The Quality Assessment of Diagnostic Accuracy Studies-2 (QUADAS-2) were utilized to assess risk of bias [11].

### Data integration and statistical analysis

Meta-analysis was conducted utilizing RevMan 5.41 (Cochrane Collaboration, London, UK) and R Statistical Software (v3.5.1, Boston, MA, USA). The diagnostic accuracy of WHCA was evaluated through summary ROC analysis. Three levels of diagnostic accuracy were analyzed: patient-based, vessel-based, and segment-based. Sensitivity and specificity were derived from ROC curves and the diagnostic performance was compared at magnetic field strengths of 1.5 T and 3.0 T. A random-effects model was employed to estimate imaging time by coil type. The inverse variance method was utilized to weight each study in the meta-analysis. Heterogeneity was indicated by I^2^, with 0% indicating no heterogeneity and 100% indicating strong heterogeneity [12]. P < 0.05 was considered statistically significant.

## Results

**Fig. 1:**
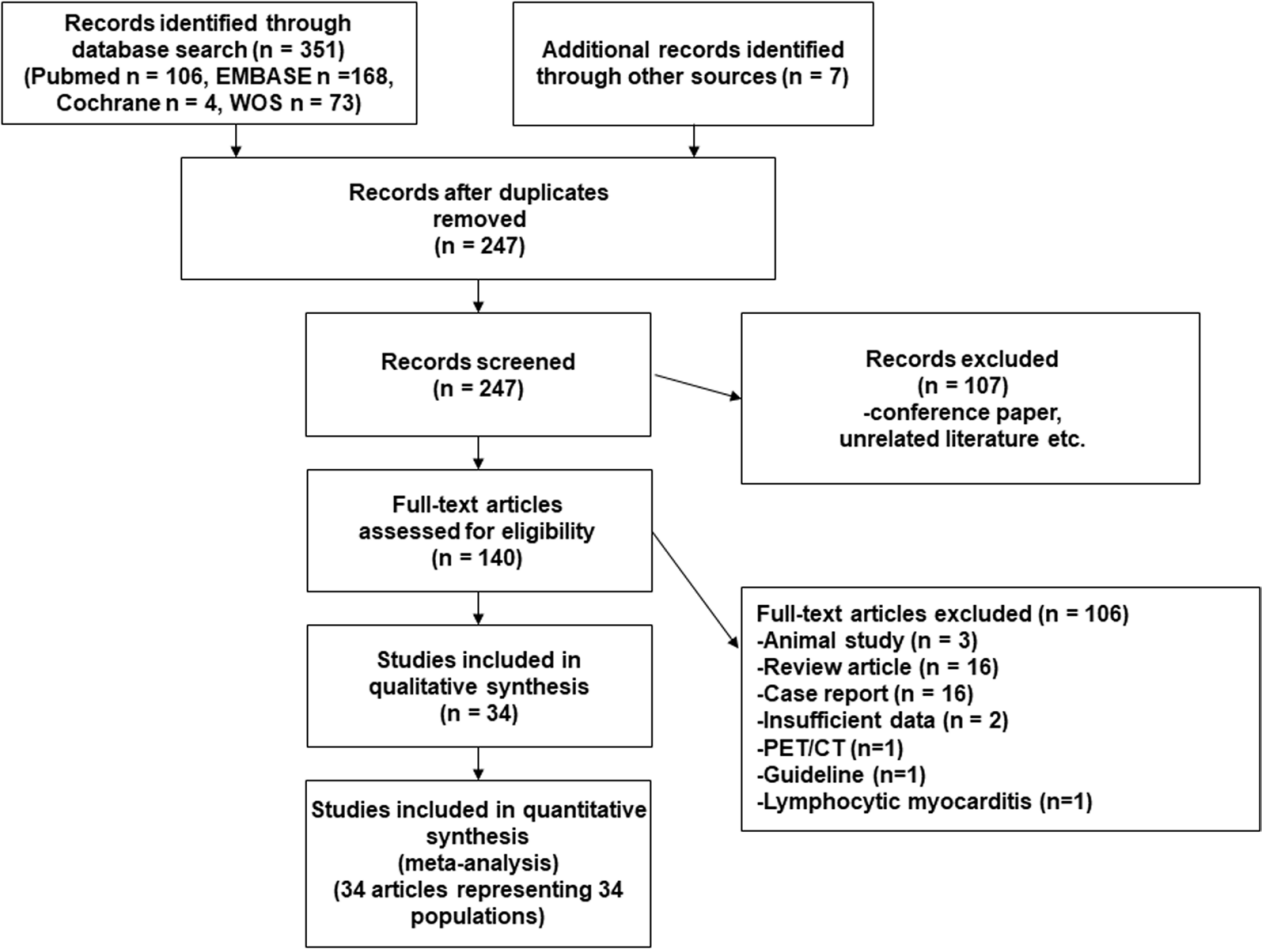
Preferred reporting items for systematic reviews and meta-analyses flow diagram

Ultimately, 34 eligible papers were selected from a pool of 140 candidate papers, and data from 1899 patients were consolidated (Fig. 1) [13–45]. The characteristics of the included studies are summarized in Table 1. The publication years of these studies ranged from 2005 to 2022; 23 of these studies utilized 1.5 T MRI technology [13–25, 28, 30, 31, 33–37, 44–46], while 11 utilized 3.0 T technology [26, 27, 29, 32, 38–43, 45]. The countries of publication were diverse, with China having 11 reports [18, 26, 32, 34, 38, 39, 41–45], Germany having 8 reports [13, 15, 19, 25, 27, 29, 31, 35], Japan having 7 reports [14, 17, 24, 28, 30, 37, 40], United Kingdom [21, 46] having two reports and various other countries such as the United States [20], Korea [16], Switzerland [36], Portugal [33], Belgium [23], and Turkey [22] having one report each. In terms of study design, one study employed a prospective multicenter design [28], while 19 employed prospective single-center designs [13–15, 17, 21, 23, 25–27, 29–32, 37–39, 43–46]. The remaining studies were retrospective in nature. The MRI sequences utilized in these studies were steady state free precession [13–25, 28, 30, 31, 33–37, 40, 44, 46] or gradient-echo [26, 27, 29, 32, 38, 39, 41–43, 45]. Twenty-three studies demonstrated the diagnostic capability of 1.5 T WHCA [13–25, 28, 30, 31, 33–37, 44, 46], three of which used contrast [31, 33, 36] (Table 2). That is, the majority of studies (87%; 20/23) performed 1.5 T WHCA imaging without contrast. Eleven studies showed diagnostic performance of 3.0 T WHCA [26, 27, 29, 32, 38–41, 43, 45], of which two studies used non-contrast imaging [29, 45]. In other words, the majority of studies (82%, 9/11) evaluated the diagnostic performance of 3.0 T with gadolinium contrast administration. In terms of sequence used, all studies at 1.5 T used SSFP (100%, 23/23). In contrast, only one study at 3.0 T used SSFP [40], while the others used GRE-based sequencing (91%, 10/11). The information of pre-pulse and fat suppression was summarized in Table 2. The definition of significant CAD was luminal narrowing ≥ 50% in almost all studies (97%, 33/34). Only one study defined significant CAD as “luminal narrowing ≥ 90%, ≥ 50% in LMT, or FFR ≤ 0.80” [33]. In terms of cardiac coils, 32-channel coils were employed in 11 reports [29–32, 37, 39, 40, 42, 44–46]. Vasodilators such as sublingual nitroglycerin were utilized in 18 studies [14, 15, 17, 18, 24, 26, 27, 29–35, 37, 40, 41, 46], and beta-blockers were utilized in 12 studies [18, 19, 22, 26, 29, 32–35, 41, 43, 46]. The results of QUADAS-2 are summarized in Additional file 1: Material S2.

**Table 1:** Characteristics of included studies

Study	Country	Study design	No of patients	Male, %	Age	Heart rate	BMI	Definition of CAD	CAD prevalence, %
Jahnke_2005	Germany	Single prospective	32	78	59 ± 10	67 ± 12	27.2 + 4.2	≥ 50%	50
Sakuma_2005	Japan	Single prospective	20	80	65 ± 12	70 ± 12	N/A	≥ 50%	60
Dewey_2006	Germany	Single prospective	129	74	64 ± 8	N/A	27.0 ± 3.5	≥ 50%	56
Kim_2006	Korea	Single	21	71	54.2	66.2 ± 14.6	N/A	≥ 50%	N/A
Sakuma_2006	Japan	Single prospective	113	87	66 ± 11	72 ± 13	N/A	≥ 50%	45
Liu_2007	China	Single	18	66	56	N/A	N/A	≥ 50%	36 coronary segments
Maintz_2007	Germany	Single	20	75	58 ± 9.7	N/A	N/A	≥ 50%	23 coronary segments
McCarthy_2007	USA	Single	33	66	57	N/A	N/A	≥ 50%	52 coronary segments
Klein_2008	UK	Single prospective	46	48	60 ± 10	73 ± 15	27.6 ± 4.1	≥ 50%	48
Oncel_2008	Turkey	Single	18	72	56.3	62 ± 10	N/A	≥ 50%	61
Pouleur_2008	Belgium	Single prospective	77	73	61 ± 14	69 ± 15	N/A	≥ 50%	22
Kunimasa_2009	Japan	Single	43	77	65 ± 13	66 ± 12	N/A	≥ 50%	77
Langer_2009	Germany	Single prospective	68	56	63.6 ± 11	64.9 ± 13	27.6 ± 3.5	≥ 50%	38
Yang_2009	China	Single prospective	62	48	61 ± 11	67 ± 7	24.1 ± 2.8	≥ 50%	55
Chen_2010	Germany	Single prospective	67	67	60 ± 10	65 ± 9	25.6 + 4.5	≥ 50%	55
Kato_2010	Japan	Multicenter prospective	127	44	67 ± 9	68 ± 12	24 + 4	≥ 50%	44
Hamdan_2011	Germany	Single prospective	110	70	65 ± 8	63 ± 8	27 ± 3.9	≥ 50%	56
Nagata_2011	Japan	Single prospective	67	58	69 ± 13	72 ± 10	23 ± 3	≥ 50%	58
Wagner_2011	Germany	Single prospective	27	13	55 ± 7	N/A	N/A	≥ 50%	67
Yang_2012	China	Single prospective	101	48	58 ± 11	66 ± 8	24 ± 3	≥ 50%	49
Bettencourt_2013	Portugal	Single	43	65	61 ± 8	65 ± 6	28.4 ± 5.43	≥ 90%, ≥ 50% in LMT, or FFR ≤ 0.80	56
Cheng_2013	China	Single	30	70	51.6 (mean)	N/A	N/A	≥ 50%	N/A
Heer_2013	Germany	Single	59	61	59 ± 13	62 ± 8	25.9 ± 3.8	≥ 50%	51
Piccini_2014	Switzerland	Single	31	68	49 ± 21	N/A	24.3 ± 4.6	≥ 50%	68
Yonezawa_2014	Japan	Single prospective	62	74	69 ± 13	73 ± 10	23 ± 3	≥ 50%	53
Yun_2014	China	Single prospective	53	86	58.7 ± 9.1	65 ± 11	N/A	≥ 50%	N/A
He_2016	China	Single prospective	39	77	57 ± 10	70 ± 7	N/A	≥ 50%	59
Namba_2016	Japan	Single retrospective	24	58	62.2 ± 16	71.0 ± 14.1	24.7 ± 3.6	≥ 50%	50
Chen_2018	China	Single	40	88	58.1 ± 10.9	64.8 ± 9.2	N/A	≥ 50%	78
Zhang_2018	China	Single	46	72	54 ± 12	67 ± 10	N/A	≥ 50%	74
Sun_2020	China	Single prospective	51	75	60.2 ± 6.7	65 ± 8	24.8 ± 2.1	≥ 50%	61
Lin_2021	China	Single prospective	45	67	58 ± 8	66 ± 9	26.1 ± 3.7	≥ 50%	73
Lu_2022	China	Single prospective	82	65	58 ± 10	68.79 ± 10.64	24.94 ± 3.78	≥ 50%	45
Nazir_2022	UK	Single prospective	45	67	62 ± 10	61 ± 8	31 ± 6	≥ 50%	42

Age, heart rate and BMI are mean ± standard deviation

*BMI* body mass index, *CAD* coronary artery disease, *FFR* fractional flow reserve, *SSFP* steady state free precession, *N/A*, not applicable

**Table 2:** Information of magnetic resonance imaging

Study	Scanner manufacturer	Sequence	Magnetic field strength (T)	Coil channels	Fat suppression	Pre-pulse	Vasodilatory premedication	Contrast agent	Beta-blocker	Scan time (min)
Jahnke_2005	Philips	SSFP	1.5	5	Yes	T2 preparation	No	No	No	12 ± 2
Sakuma_2005	Philips	SSFP	1.5	5	Yes	T2 preparation	Yes	No	No	13.8 ± 3.8
Dewey_2006	Siemens Medical Solutions	SSFP	1.5	12	Yes	None	Yes	No	No	N/A
Kim_2006	Philips Medical Systems	SSFP	1.5	Synergy cardiac coil	Yes (SPIR)	T2 preparation	N/A	N/A	N/A	9.3 ± 2.1
Sakuma_2006	Philips	SSFP	1.5	5	Yes	T2 preparation	Yes	No	No	12.9 ± 4.3
Liu_2007	GE Healthcare	SSFP	1.5	6	Yes	T2 preparation	Yes	No	Yes	N/A
Maintz_2007	Philips	SSFP	1.5	5	Yes (SPIR)	T2 preparation	No	No	Yes	14
McCarthy_2007	Siemens Medical Solutions	SSFP	1.5	12	Yes	N/A	No	No	No	45
Klein_2008	Philips	SSFP	1.5	5	Yes	T2 preparation	No	No	No	6.3 ± 1.6
Oncel_2008	Siemens	SSFP	1.5	N/A	Yes	T2 preparation	No	No	Yes	13
Pouleur_2008	Philips	SSFP	1.5	5	Yes	T2 preparation	No	No	No	20 ± 4
Kunimasa_2009	Philips	SSFP	1.5	5	N/A	T2 preparation	Yes	No	No	9 ± 3.1
Langer_2009	Philips	SSFP	1.5	5	Yes (SPIR)	T2 preparation	No	No	No	N/A
Yang_2009	Siemens	GRE	3	12	Yes	Inversion recovery	Yes	Yes	Yes	9 ± 1.9
Chen_2010	Siemens	GRE	3	12	Yes	Inversion recovery	Yes	Yes	No	9.6 ± 3.2
Kato_2010	Philips	SSFP	1.5	5	Yes	T2 preparation	No	No	No	9.5 ± 3.5
Hamdan_2011	Philips	GRE	3	32	Yes	T2 preparation	Yes	No	Yes	17 ± 4.7
Nagata_2011	Philips	SSFP	1.5	32	Yes	T2 preparation	Yes	No	No	6.2 ± 2.8
Wagner_2011	Siemens	SSFP	1.5	32	Yes	T2 preparation	Yes	Yes	No	9.1 ± 2
Yang_2012	Siemens	GRE	3	32	N/A	Inversion recovery	Yes	Yes	Yes	7 ± 1.8
Bettencourt_2013	Siemens	SSFP	1.5	12	Yes	T2 preparation	Yes	Yes	Yes	17.9 ± 4.6
Cheng_2013	General Electric Healthcare Technologies	SSFP	1.5	8	Yes	T2 preparation	Yes	No	Yes	N/A
Heer_2013	GE	SSFP	1.5	8	Yes	T2 preparation	Yes	No	Yes	14.3 ± 6.2
Piccini_2014	Siemens	SSFP	1.5	30	Yes	T2 preparation	N/A	Yes	No	7.8 ± 1.9
Yonezawa_2014	Philips	SSFP	1.5	32	Yes	T2 preparation	Yes	No	No	6.8 ± 2.6
Yun_2014	Siemens	GRE	3	12	Yes (SPIR)	Inversion recovery	No	Yes	No	10.3 ± 2.5
He_2016	Siemens	GRE	3	32	Yes	Inversion recovery	No	Yes	No	7.8 ± 0.8
Namba_2016	Philips	SSFP	3	32	Yes (SPIR)	T2 preparation	Yes	Yes	No	278 ± 43 s
Chen_2018	Siemens	GRE	3	12	Yes	Inversion recovery	Yes	Yes	Yes	9.5 ± 3.1
Zhang_2018	Siemens	GRE	3	32	Yes	Inversion recovery	N/A	Yes	No	10.4 ± 3.2
Sun_2020	Siemens	GRE	3	12	Yes	Inversion recovery		Yes	Yes	9.5 ± 3.1
Lin_2021	Philips	SSFP	1.5	32	Yes (SPIR)	T2 preparation	No	No	No	10.2 ± 2.4
Lu_2022	Philips	GRE	3	32	N/A	T2 preparation	No	No	No	7.88 ± 2.78
Nazir_2022	Siemens	SSFP	1.5	32	Yes (SPIR)	T2 preparation	Yes	No	Yes	10.4 ± 2.1

*BMI* body mass index, *CAD* coronary artery disease, *GRE* gradient echo, *SSFP* steady state free precession, *SPIR* spectral presaturation with inversion recovery, *N/A* not applicable

### Diagnostic accuracy of WHCA—1.5 T vs. 3.0 T

**Fig. 2:**
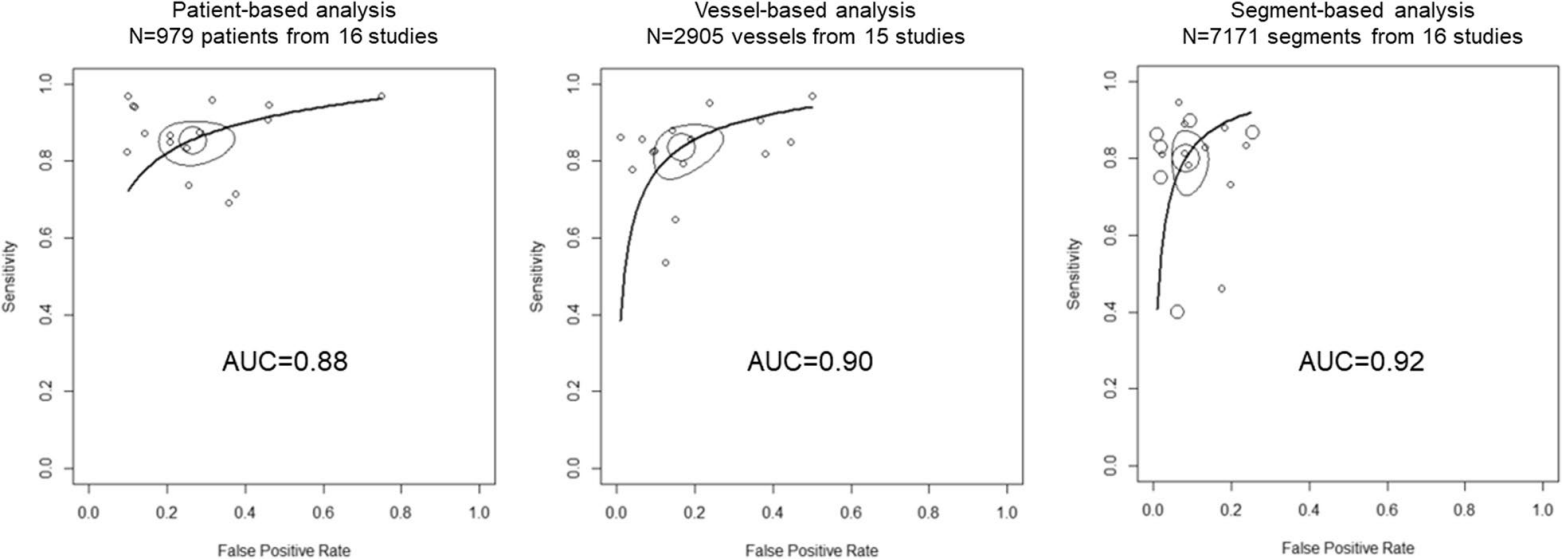
Summary ROC curve of 1.5 T whole-heart coronary MRA

**Fig. 3:**
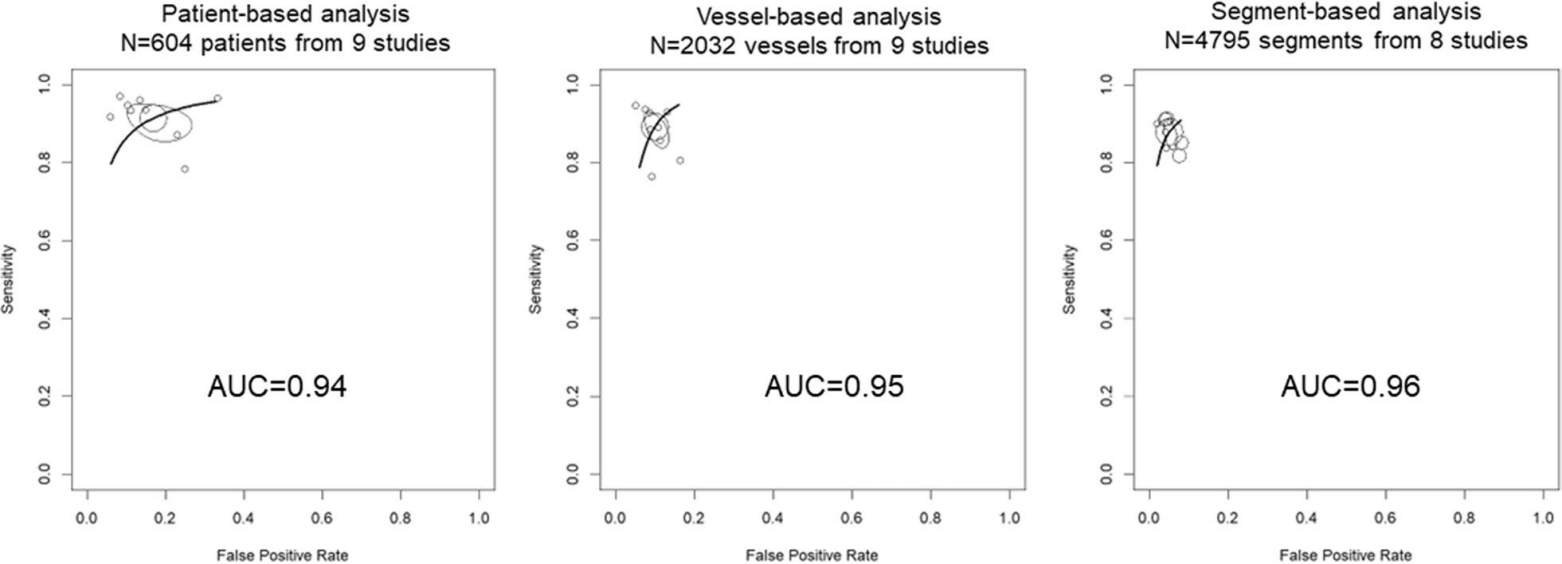
Summary ROC curve of 3.0 T whole-heart coronary MRA

Figure 2 illustrates the summary receiver operating characteristic analysis of the ability of 1.5 T WHCA to detect significant stenosis when the gold standard is significant stenosis on X-ray coronary angiography. The area under the curve was 0.88 for patient-based analysis (N = 979 patients from 16 studies), 0.90 for vessel-based analysis (N = 2905 vessels from 15 studies), and 0.92 for segment-based analysis (N = 7171 segments from 16 studies). Figure 3 illustrates the summary receiver operating characteristic analysis of the ability of 3.0 T WHCA to detect significant stenosis. The area under the curve was 0.94 for patient-based analysis (N = 604 patients from 9 studies) and 0.95 for vessel-based analysis (N = 2032 vessels from 9 studies), and 0.96 for segment-based analysis (N = 4795 segments from 8 studies). Table 3 summarizes the sensitivity and specificity calculated from the receiver operating characteristic curves. The 3.0 T WHCA technique had significantly higher sensitivity in the segment-based analysis compared to 1.5 T (0.88, 95% confidence interval (CI) 0.84–0.91 vs. 0.80, 95% CI 0.72–0.86, P = 0.04). The individual diagnostic performance of each study is summarized in Additional file 1: Materials S3–S5.

**Table 3:** Sensitivity and specificity of whole heart coronary MRA for the detection of significant coronary stenosis on X-ray coronary angiogram

	1.5 T WHCA (N = 23 studies)	3.0 T WHCA (N = 11 studies)	P-value*
Patient-based analysis			
Sensitivity	0.86 (0.80–0.90)	0.91 (0.87–0.94)	0.10
Specificity	0.73 (0.65–0.81)	0.83 (0.75–0.89)	0.06
Vessel-based analysis			
Sensitivity	0.84 (0.77–0.88)	0.89 (0.85–0.92)	0.13
Specificity	0.83 (0.75–0.89)	0.90 (0.83–0.92)	0.09
Segment-based analysis			
Sensitivity	0.80 (0.72–0.86)	0.88 (0.84–0.91)	0.04
Specificity	0.92 (0.87–0.95)	0.95 (0.94–0.96)	0.25

Data are weighted mean ± 95% confidence interval

*WHCA* whole heart coronary magnetic resonance imaging, *MRA* magnetic resonance imaging

*P-value represents the significance of difference between 1.5 and 3.0 Tesla WHCA

### Sub-analysis of diagnostic performance of WHCA

As previously stated, 1.5 T WHCA is typically performed without the use of gadolinium contrast, while 3.0 T WHCA is typically performed with contrast administration. Keeping this in mind, we conducted a comparison of the diagnostic performance of non-contrast 1.5 T WHCA and contrast-enhanced 3.0 T WHCA. The 3.0 T WHCA technique demonstrated significantly higher specificity compared to the non-contrast enhanced 1.5 T WHCA on a patient-based analysis (Table 4). Additionally, we performed a subgroup analysis based on the presence or absence of drug use and geographical difference (Asian and Western countries). There were no differences in diagnostic performance on a patient-based analysis were observed in the use of vasodilators and beta-blockers (Tables 5, 6) or comparison between Asian and Western countries (Table 7). However, in the vessel-based analysis, the sensitivity of studies using the vasodilator was lower than those not using it (P = 0.03) (Table 5). Mean heart rate in the study with beta-blocker administration was 64.6 bpm (95% CI 63.9–65.3 bpm) and in the study without beta-blocker administration mean heart rate was 68.6 bpm (95% CI 67.9–69.2 bpm), a significant difference was found between the two groups (P < 0.001). There was a significant difference in heart rate between trials with and without beta-blockers, but no difference in diagnostic performance. In the regional analysis, the body mass index (BMI) reported in Western countries was significantly higher than that in Asian countries (27.0 kg/m^2^, 95% CI 26.6–27.3 vs. 23.7 kg/m^2^, 95% CI 23.3–24.1, P < 0.001).

**Table 4:** Comparison of diagnostic performance of non-contrast 1.5 T WHCA and contrast-enhanced 3.0 T WHCA for the detection of significant CAD

	Non-contrast 1.5 T WHCA (N = 20 studies)	Contrast-enhanced 3.0 T WHCA (N = 9 studies)	P-value
Patient-based analysis			
Sensitivity	0.86 (0.80–0.90)	0.92 (0.87–0.96)	0.07
Specificity	0.74 (0.64–0.82)	0.87 (0.80–0.92)	0.02
AUC	0.88	0.94	N/A
Vessel-based analysis			
Sensitivity	0.84 (0.77–0.90)	0.91 (0.86–0.94)	0.07
Specificity	0.86 (0.77–0.92)	0.91 (0.89–0.93)	0.20
AUC	0.91	0.95	N/A
Segment-based analysis			
Sensitivity	0.82 (0.75–0.88)	0.88 (0.85–0.91)	0.1
Specificity	0.93 (0.88–0.95)	0.95 (0.94–0.96)	0.28
AUC	0.93	0.95	N/A

Data are weighted mean ± 95% confidence interval

*AUC* area under the curve, *CAD* coronary artery disease, *WHCA* whole heart coronary magnetic resonance angiography

**Table 5:** A comparative analysis of sensitivity, specificity, and AUC between WHCA with and without vasodilators

	1.5 T WHCA with vasodilator (N = 12 studies)	1.5 T WHCA without vasodilator (N = 9 studies)	P-value
Patient-based analysis			
Sensitivity	0.86 (0.80–0.90)	0.88 (0.75–0.95)	0.85
Specificity	0.76 (0.69–0.82)	0.65 (0.58–0.82)	0.11
AUC	0.88	0.86	N/A
Vessel-based analysis			
Sensitivity	0.81 (0.74–0.87)	0.91 (0.83–0.96)	0.03
Specificity	0.87 (0.79–0.92)	0.73 (0.50–0.88)	0.17
AUC	0.9	0.92	N/A
Segment-based analysis			
Sensitivity	0.84 (0.79–0.87)	0.80 (0.65–0.89)	0.54
Specificity	0.92 (0.87–0.96)	0.93 (0.85–0.96)	0.78
AUC	0.85	0.93	N/A

Data are weighted mean ± 95% confidence interval

*AUC* area under the curve, *WHCA* whole heart coronary magnetic resonance angiography

**Table 6:** A comparative analysis of sensitivity, specificity, and AUC between WHCA with and without beta-blockers

	1.5 T WHCA with beta-blocker (N = 7 studies)	1.5 T WHCA without beta-blocker (N = 15 studies)	P-value
Patient-based analysis			
Sensitivity	0.91 (0.81–0.96)	0.84 (0.78–0.89)	0.14
Specificity	0.69 (0.57–0.78)	0.75 (0.65–0.83)	0.39
AUC	0.89	0.87	N/A
Vessel-based analysis			
Sensitivity	0.85 (0.77–0.91)	0.83 (0.75–0.89)	0.69
Specificity	0.82 (0.70–0.89)	0.85 (0.75–0.91)	0.63
AUC	0.89	0.9	N/A
Segment-based analysis			
Sensitivity	0.84 (0.77–0.89)	0.78 (0.66–0.87)	0.33
Specificity	0.91 (0.83–0.95)	0.93 (0.87–0.96)	0.6
AUC	0.9	0.93	N/A

Data are weighted mean ± 95% confidence interval

*AUC* area under the curve, *WHCA* whole heart coronary magnetic resonance angiography

**Table 7:** A comparative analysis of sensitivity, specificity, and AUC between Asian and Western countries

	1.5 T WHCA (Asian countries) (N = 10 studies)	1.5 T WHCA (Western countries) (N = 13 studies)	P-value
Patient-based analysis			
Sensitivity	0.86 (0.80–0.90)	0.84 (0.75–0.90)	0.66
Specificity	0.89 (0.78–0.95)	0.81 (0.62–0.78)	0.17
AUC	0.9	0.84	N/A
Vessel-based analysis			
Sensitivity	0.84 (0.77–0.89)	0.81 (0.70–0.88)	0.58
Specificity	0.84 (0.74–0.90)	0.79 (0.71–0.85)	0.35
AUC	0.91	0.86	N/A
Segment-based analysis			
Sensitivity	0.85 (0.81–0.88)	0.76 (0.61–0.86)	0.17
Specificity	0.93 (0.85–0.97)	0.91 (0.86–0.94)	0.59
AUC	0.87	0.92	N/A

Data are weighted mean ± 95% confidence interval

*AUC* area under the curve, *WHCA* whole heart coronary magnetic resonance angiography

## Discussion

The main findings of this study are as follows: Receiver operating characteristic analysis showed that the 3 T field strength was superior in detecting significant coronary arteries compared to the 1.5 T. In addition, a direct comparison of non-contrast 1.5 T WHCA and contrast-enhanced 3.0 T WHCA was performed as a practical comparison, with the latter showing significantly higher specificity on a patient-based analysis. Subgroup analyses also showed no significant difference in diagnostic performance of 1.5 T WHCA in terms of the use of vasodilators and beta-blockers on a patient-based analysis. Although BMI was higher in Western patients compared to Asian patients, there was no difference in the diagnostic performance of the 1.5 T WHCA. These results suggest that WHCA is useful for noninvasive detection of significant CAD.

WHCA is well-established as a non-invasive method for the screening of CAD and possesses a number of advantages, such as the absence of ionizing radiation exposure, decreased susceptibility to calcification, and the lack of a requirement for contrast agent administration at 1.5 T. However, there is limited evidence for its clinical utility. To date, numerous studies have utilized X-ray coronary angiography as the gold standard, yet the majority of these studies have been conducted on small patient populations at a single institution. The only prospective, multi-center study was conducted in Japan and reported a sensitivity of 88% and specificity of 72% for 1.5 T WHCA [28]. Unfortunately, there have been no further multi-center studies since. Additionally, 3 T MR is often performed utilizing the gradient echo method, which necessitates the administration of a contrast agent [26, 27, 32, 38–43]. After contrast agent administration, 3 T WHCA provides a higher signal-to-noise ratio compared to 1.5 T WHCA and has been reported to have high diagnostic performance for the detection of coronary artery stenosis. However, one of the major advantages of MRI, the lack of requirement for contrast agent administration, is lost with 3 T WHCA. Recently, attempts have been made to perform non-contrast 3 T WHCA imaging, with promising results, but the number of reports on this technique is limited [29, 45]. The administration of a gadolinium contrast agent is necessary for 3 T WHCA due to the difficulties in using SSFP caused by SAR limitations and banding artifacts resulting from B1 inhomogeneity. Therefore, GRE is generally the preferred imaging sequence, but in order to achieve sufficient vascular contrast, the administration of gadolinium contrast is required [10]. As the imaging methods and diagnostic accuracy of 1.5 T and 3 T coronary WHCA are fundamentally different, separate meta-analyses are required. However, meta-analyses reported to date have included a mixture of 1.5 T and 3 T WHCA systems [7, 8]. Therefore, the primary objective of the present meta-analysis was to compare the diagnostic performance of WHCA with two different magnetic field strengths. Our results demonstrated that 3 T provided superior diagnostic performance when compared to 1.5 T, however, the number of reports regarding 3 T WHCA was small and there was a large bias in the countries and facilities where the studies were conducted (8/11 reports from China), making it difficult to generalize the obtained data. Further evidence accumulation and large-scale, prospective, multi-center studies are needed in the future to further investigate the diagnostic performance of 3 T MR. The clinical significance of the difference in diagnostic performance between 1.5 and 3.0 T WHCA is debatable. While 3.0 T WHCA exhibits slightly superior diagnostic performance, its major disadvantage of requiring the administration of gadolinium-based contrast agents negates its advantages over coronary CTA. Therefore, 1.5 T WHCA, which has unique benefits such as no radiation exposure and no need for gadolinium-based contrast administration, may be more clinically practical.

Another significant clinical query revolves around the necessity of nitroglycerin or beta-blockers in WHCA. Subgroup analyses of trials including and excluding both medications demonstrated comparable diagnostic performance in patient-based analyses, irrespective of drug usage (Tables 5, 6). Notably, there was no disparity in diagnostic performance, despite lower heart rates observed in studies employing beta-blockers. This could be attributed to the minimal absolute difference in heart rates (64.6 bpm vs. 68.6 bpm). Furthermore, although no distinctions were found in patient-based or segment-based analyses concerning vasodilator use, the sensitivity of studies employing vasodilators was lower than those that did not, as revealed by the vessel-based analysis (0.81 vs 0.91, P = 0.03, Table 5). Although the exact cause remains unclear, the vessel-based analysis exhibited higher AUC values for both groups, with an AUC of 0.90 for studies utilizing vasodilators and an AUC of 0.92 for studies without vasodilators. Given the trade-off relationship between sensitivity and specificity, the AUC does not appear to indicate a substantial disparity in diagnostic performance between studies with and without vasodilator use.

In addition, it is posited that coronary MRA is utilized by numerous institutions in Asian countries, with fewer employing it in Western countries. In light of this, we conducted a subgroup analysis of 1.5 T WHCA, taking into account the possibility of reduced diagnostic performance in larger patients due to their larger body size in Western countries (3.0 T studies could not be analyzed due to their small number). The findings indicated that BMI was significantly higher in patients from Western countries, but no significant differences in diagnostic performance were discerned between the two groups (Table 7). This suggests that WHCA can maintain its diagnostic efficacy even in patients with larger body mass.

The assessment of diagnostic efficiency between WHCA and coronary CT is a highly pertinent clinical inquiry. Nevertheless, there are few studies that have directly compared the two modalities. For instance, it has been demonstrated that WHCA is more diagnostically reliable than coronary CT in highly calcified segments of coronary arteries with calcification scores of 100 or above [18]. Conversely, other studies have found that 3 T WHCA has comparable diagnostic accuracy to CTA [43, 47]. Although such small-scale studies are dispersed, there is a lack of large-scale, coherent data, and it is challenging to statistically validate the comparison in this meta-analysis. Regardless, it is incontrovertible that coronary CT is the primary test for screening for CAD, owing to its spatial resolution, imaging duration, and reported high diagnostic accuracy. WHCA may serve as a viable alternative for patients for whom coronary CT cannot be performed, such as those with iodine allergies. Additionally, it should be utilized assertively in young patients who should not be exposed to radiation, female patients, and patients with coronary artery malformations [48] or coronary aneurysms in Kawasaki disease [49], which can be adequately evaluated with MRI resolution. Further accumulation of evidence on these points is also desirable.

Recent advancements in high-speed imaging techniques, such as compressed sensing, have the potential to shorten the imaging time for WHCA [50]. Furthermore, advancements in imaging techniques utilizing artificial intelligence are anticipated to enhance spatial resolution and decrease noise, thereby improving the image quality of WHCA. Deep learning reconstruction techniques have been used to improve the contrast-to-noise ratio and image quality of high-resolution WHCA [51]. A volunteer study has also demonstrated the potential of deep learning reconstruction for WHCA with sub-millimeter isotropic resolution at 3T [52]. These innovations in imaging technology are expected to further enhance the diagnostic accuracy of WHCA.

### Limitations

First, many of the studies analysed were single centre studies with a limited number of cases, and the variability in study results cannot be ruled out. Prospective multicentre studies that include a larger number of patients are desirable. Second, we performed several subgroup analyses, but the number of included studies may be too small to produce statistically valid results.

## Conclusions

The diagnostic performance of WHCA was deemed satisfactory, with contrast-enhanced 3.0 T WHCA exhibiting higher specificity compared to non-contrast-enhanced 1.5 T WHCA in a patient-based analysis. No significant differences in diagnostic performance were observed on a patient-based analysis based on the use of vasodilators, beta-blockers, or geographical regions (Asian and Western countries). Further large multicentre studies are imperative to facilitate the global adoption of WHCA.

## Supplementary Information

**Additional file 1: Material S1.** PubMed 106, WOS 73, Cochrane 4, EMBASE. **Material S2.** QUADAS-2. **Material S3.** Sensitivity and Specificity of WHCA on patient-based analysis. **Material S4.** Sensitivity and Specificity of WHCA on vessel-based analysis. **Material S5.** Sensitivity and Specificity of WHCA on segment-based analysis.

## Data Availability

The datasets analysed in the current study are available from the corresponding author upon reasonable request.

## Abbreviations

BMI: Body mass index
CAD: Coronary artery disease
CI: Confidence interval
CT: Computed tomography
GRE: Gradient echo
MRI: Magnetic resonance imaging
ROC: Receiver operating characteristics
SSFP: Steady state free precession
WHCA: Whole heart coronary magnetic resonance angiography
