# Supplementary material for: Diagnostic accuracy of whole heart coronary magnetic resonance angiography: a systematic review and meta-analysis
Source: J Cardiovasc Magn Reson. 2023 Jun 26;25:36. doi: 10.1186/s12968-023-00949-6 (PMC10291762; doi:10.1186/s12968-023-00949-6)

**Additional material S1**

*PubMed 1**06*

*(whole heart coronary magnetic resonance angiography [title]OR whole heart coronary MRA[title]) AND*

*(coronary artery disease [title]OR chronic coronary syndrome [title]OR myocardial infarction [title]OR angina pectoris[title])AND (diagnostic accuracy[title] OR diagnostic performance[title] OR sensitivity[title] OR specificity[title])*

*WOS 73*

*#1 TI=(whole heart coronary magnetic resonance angiography [title]OR whole heart coronary MRA[title])*

*#2 TS=(coronary artery disease [title]OR chronic coronary syndrome [title]OR myocardial infarction [title]OR angina pectoris[title])*

*#3 TS=(diagnostic accuracy[title] OR diagnostic performance[title] OR sensitivity[title] OR specificity[title])*

*#4 #1 AND #2 AND #3*

*Cochrane 4*

*#1 whole heart coronary magnetic resonance angiography:ti OR whole heart coronary MRA:ti*

*#2 coronary artery disease:ti OR chronic coronary syndrome:ti OR myocardial infarction:ti OR angina pectoris:ti*

*#3 diagnostic accuracy:ti OR diagnostic performance:ti OR sensitivity:ti OR specificity:ti*

*#4 #1 AND #2 AND #3*

*EMBASE*

*QUICK SEARCH: 168*

*TITLE: (whole heart coronary magnetic resonance angiography OR whole heart coronary MRA) AND*

*(coronary artery disease OR chronic coronary syndrome OR myocardial infarction OR angina pectoris)AND (diagnostic accuracy OR diagnostic performance OR sensitivity OR specificity)*

**Additional material S2. QUADAS-2**

|  | **RISK OF BIAS** | | | | **APPLICABILITY CONCERNS** | | |
| --- | --- | --- | --- | --- | --- | --- | --- |
|  | **Patient selection** | **Index test** | **Reference standard** | **Flow and timing** | **Patient selection** | **Index test** | **Reference standard** |
| **Bettencourt_2013** | **H** | **L** | **L** | **L** | **H** | **L** | **L** |
| **Chen_2010** | **L** | **L** | **L** | **L** | **L** | **L** | **L** |
| **Chen_2018** | **H** | **L** | **L** | **L** | **H** | **L** | **L** |
| **Cheng_2013** | **H** | **L** | **L** | **L** | **H** | **L** | **L** |
| **Dewey_2006** | **L** | **L** | **L** | **L** | **L** | **L** | **L** |
| **Hamdan_2011** | **L** | **L** | **L** | **L** | **L** | **L** | **L** |
| **He_2016** | **L** | **L** | **L** | **L** | **L** | **L** | **L** |
| **Heer_2013** | **H** | **L** | **L** | **L** | **H** | **L** | **L** |
| **Jahnke_2005** | **L** | **L** | **L** | **L** | **L** | **L** | **L** |
| **Kato_2010** | **L** | **L** | **L** | **L** | **L** | **L** | **L** |
| **Kim_2006** | **H** | **L** | **L** | **L** | **H** | **L** | **L** |
| **Klein_2008** | **L** | **L** | **L** | **L** | **L** | **L** | **L** |
| **Kunimasa_2009** | **H** | **L** | **L** | **L** | **H** | **L** | **L** |
| **Langer_2009** | **L** | **L** | **L** | **L** | **L** | **L** | **L** |
| **Lin_2021** | **L** | **L** | **L** | **L** | **L** | **L** | **L** |
| **Liu_2007** | **H** | **L** | **L** | **L** | **H** | **L** | **L** |
| **Lu_2022** | **L** | **L** | **L** | **L** | **L** | **L** | **L** |
| **Maintz_2007** | **H** | **L** | **L** | **L** | **H** | **L** | **L** |
| **McCarthy_2007** | **H** | **L** | **L** | **L** | **H** | **L** | **L** |
| **Nagata_2011** | **L** | **L** | **L** | **L** | **L** | **L** | **L** |
| **Nazir_2022** | **L** | **L** | **L** | **L** | **L** | **L** | **L** |
| **Namba_2016** | **H** | **L** | **L** | **L** | **H** | **L** | **L** |
| **Oncel_2008** | **H** | **L** | **L** | **L** | **H** | **L** | **L** |
| **Piccini_2014** | **H** | **L** | **L** | **L** | **H** | **L** | **L** |
| **Pouleur_2008** | **L** | **L** | **L** | **L** | **L** | **L** | **L** |
| **Sakuma_2005** | **L** | **L** | **L** | **L** | **L** | **L** | **L** |
| **Sakuma_2006** | **L** | **L** | **L** | **L** | **L** | **L** | **L** |
| **Sun_2020** | **L** | **L** | **L** | **L** | **L** | **L** | **L** |
| **Wagner_2011** | **L** | **L** | **L** | **L** | **L** | **L** | **L** |
| **Yang_2009** | **L** | **L** | **L** | **L** | **L** | **L** | **L** |
| **Yang_2012** | **L** | **L** | **L** | **L** | **L** | **L** | **L** |
| **Yonezawa_2014** | **L** | **L** | **L** | **L** | **L** | **L** | **L** |
| **Yun_2014** | **L** | **L** | **L** | **L** | **L** | **L** | **L** |
| **Zhang_2018** | **H** | **L** | **L** | **L** | **H** | **L** | **L** |

L: low risk, H: high risk

**Additional material S3. Sensitivity and Specificity of WHCA on patient-based analysis**


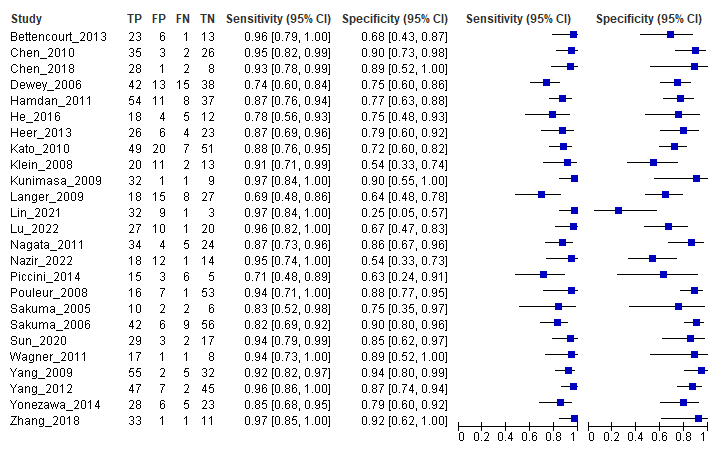


**Additional material S4. Sensitivity and Specificity of WHCA on vessel-based analysis**


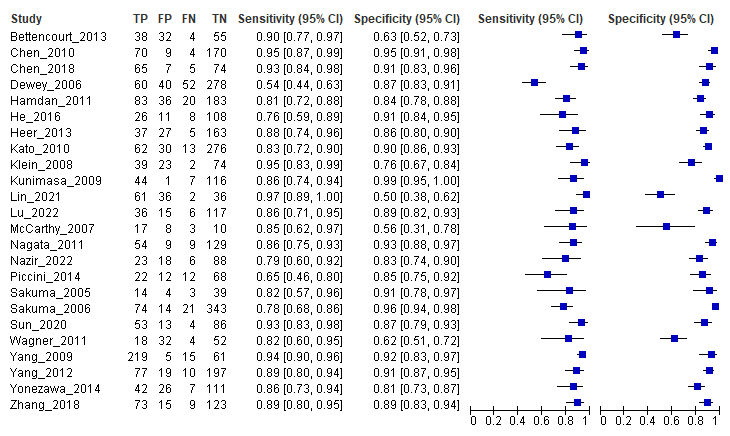


**Additional material S5. Sensitivity and Specificity of WHCA on segment-based analysis**


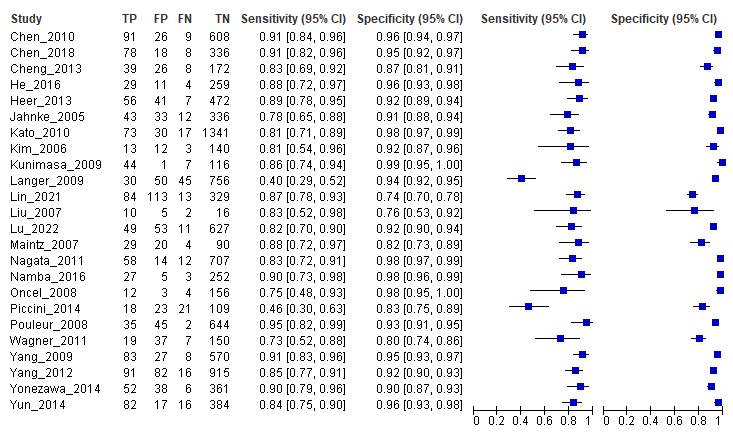

Supplement: Supplementary file 1 — Additional file 1: Material S1. PubMed 106, WOS 73, Cochrane 4, EMBASE. Material S2. QUADAS-2. Material S3. Sensitivity and Specificity of WHCA on patient-based analysis. Material S4. Sensitivity and Specificity of WHCA on vessel-based analysis. Material S5. Sensitivity and Specificity of WHCA on segment-based analysis. [file 12968_2023_949_MOESM1_ESM.docx]
